# Saliva and Meningococcal Transmission

**DOI:** 10.3201/eid0910.030444

**Published:** 2003-10

**Authors:** Hilary J. Orr, Steve J. Gray, Mary Macdonald, James M. Stuart

**Affiliations:** * Health Protection Agency (South West), Gloucester, England, UK; † Meningococcal Reference Unit, Manchester, England, UK; ‡ County Hospital, Hereford, England, UK

## Abstract

*Neisseria meningitidis* carriage was compared in swab specimens of nasopharynx, tonsils, and saliva taken from 258 students. We found a higher yield in nasopharyngeal than in tonsillar swabs (32% vs. 19%, p<0.001). Low prevalence of carriage in saliva swabs (one swab [0.4%]) suggests that low levels of salivary contact are unlikely to transmit meningococci.

Invasive meningococcal disease has a high case-fatality rate and an immediate risk of further cases among household contacts. Public health measures therefore include prompt identification of contacts for chemoprophylaxis (*1*). One question that commonly arises is whether salivary contact through sharing cups or glasses is an indication for prophylaxis, but the evidence base to inform an answer is weak, and national guidelines are inconsistent (*1*,*2*). Although saliva is thought to inhibit meningococcal growth (*3*), carriage rates in saliva are not known, and swabs to detect carriage are usually taken from tonsils or nasopharynx (*4*–*6*). We compared meningococcal isolation rates in swabs of saliva (front of mouth), tonsils, and nasopharynx.

We recruited volunteers among students from two colleges in Hereford, England. After giving written consent, students completed a short questionnaire on age, sex, smoking, recent antimicrobial drug use, and meningococcal vaccine status. Three sterile, dry, cotton-tipped swabs were used to take samples from each volunteer: one from the nasopharynx (through the mouth and swept up behind the uvula), one from both tonsils, and one swab of saliva between the lower gum and lips. Swabs were plated directly onto a selective culture medium primarily designed for the isolation of pathogenic *Neisseria* species (modified New York City base containing vancomycin, colistin, and trimethoprim), prepared by Taunton Media Services, UK (*7*). The plates were transported to Hereford Public Health Laboratory, where they were spread once from the primary inoculum and incubated in 7% CO_2_ at 37°C for 48 h. Putative *Neisseria* species isolated were sent to the Meningococcal Reference Unit, Manchester Public Health Laboratory, for *Neisseria meningitidis* confirmation and serologic phenotypic characterization. Data were entered into the computer using Excel (Microsoft Corp., Redmond, WA). Carriage rates by site were compared with McNemar’s test and by risk factor using chi-square tests. Ethical approval was obtained from the Public Health Laboratory Service Ethics Committee and Herefordshire District Ethics Committee.

Of the 258 participants, 90 (35%) were identified as carrying *Neisseria meningitidis* from one or more sites. The site with the highest yield was the nasopharynx (32.2%), whereas tonsillar carriage was 19.4% (Table). One (0.4%) of the 258 saliva swab specimens was positive. No one had positive specimens from all three sites, and the person with the positive saliva swab had negative swabs from the other two sites. Differences in carriage rates between the nasopharynx and tonsils and between the nasopharynx and saliva were statistically significant (p<0.001 in both cases).

**Table:** Carriage of *Neisseria meningitidis* by site^a^

Site of swab	One site positive	Two sites positive	Three sites positive	Total positive	Overall carriage %
Nasopharynx	39	44	0	83	32.2
Tonsils	6	44	0	50	19.4
Saliva	1	0	0	1	0.4

^a^(n=258). ^1^H.O. was responsible for recruiting students, obtaining specimens, swabs, and drafting the paper with J.S.; S.G. and M.M. were responsible for microbiologic processing and analysis; and J.S. designed the study and drafted the paper with H.O. All authors contributed to the final draft.

The predominant serogroup among carried strains was B. No serogroup C strains were identified. Of the 44 carriers with positive swabs from both nasopharynx and tonsils, each pair of isolates was considered to be phenotypically indistinguishable by serogroup, serotype, and sero-subtype. In three of these pairs, one isolate expressed serogroup B, and the paired isolate could not be serogrouped but had identical serotype and sero-subtype.

Of the 258 participants, 116 (45%) were men, and 142 were women. Most (86%) were 18 to 21 years of age. Carriage rates were higher among men than women (54/116 vs. 36/142, p<0.001), and among smokers than nonsmokers (49/90 vs. 51/168, p<0.001). Carriage rates were similar when persons were stratified by age, meningococcal vaccination status, and recent antimicrobial drug use. Although duplicate swabs from the nasopharynx sometimes yield different meningococcal strains (*3*), none of the paired isolates in this study were distinguishable by phenotype.

The yield of meningococci from nasopharyngeal swabs was nearly twice as high as that from tonsillar swabs. Previous researchers have found a lower sensitivity of nasopharyngeal swabs taken through the nose using small cotton-tipped wire swabs compared to tonsillar swabs taken using larger cotton-tipped swabs (*5*,*6*). No previous studies have compared yields from the nasopharynx and tonsils with those from the same type of swabs taken through the mouth. The carriage rate was higher than expected for this age group (*4*), suggesting that we had efficient swabbing and microbiologic techniques. We suggest that throat swabs to detect meningococcal carriage should always be taken from the nasopharynx (through the mouth whenever practical) and not from the tonsils.

The very low isolation rate from saliva swabs suggests that low levels of salivary contact are unlikely to transmit meningococci (*1*). This observation is supported by results of a case-control study among university students that found no association between meningococcal acquisition and sharing of glasses or cigarettes (*8*). On the basis of this evidence, we propose that guidelines for public health management of meningococcal disease should not include low-level salivary contact (e.g., sharing drinks) with a case-patient as an indication for chemoprophylaxis.
